# Cases of Local Affections of the Leg, Successfully Treated by Plaster-Strapping

**Published:** 1821-06

**Authors:** C. W. Smerdon

**Affiliations:** Surgeon.


### *Cases of local Affections of the Leg, successfully treated by Plaster-Strapping.* By C. W. Smerdon, Surgeon.

I AM not aware that any other author than Sterne has dedicated a chapter exclusively to the subject of hobby-horses: still we are not to infer, from this want of respect in the antients for such favourite animals, that they were unknown to them, but rather that they were too common to excite particular attention: indeed, taking it for granted that human nature is the same now as it ever was, we need make no scruples of conscience about determining that they were in use from the earliest periods, and will be so to the end of time.

Among the professors of the healing art, hobby-horses are, and have been, very common,—and very serviceable, too, if not to the public who have frequently been kicked by them, they have been to their riders, although sometimes they brake down with them when ridden too hard, and were then totally incapacitated from rising any more. Since the publication of Mr. Abernethy's excellent work on Local and Constitutional Diseases, and the dissemination of the no less excellent Lectures of the late Dr. Curry, disorders of the biliary functions and of the digestive organs, have been quite as hobby-horsical among the faculty, as were the spasm of Cullen and the splendid doctrines of Brown.

It is quite natural that the promulgator of a new hobby for public use should ride it himself; and, if Mr. Abernethy has ridden his on all occasions, and sometimes too in a hard trot, the generality of people will readily excuse him for it, because the hobby is his own, and has been upon the whole of considerable benefit to them: but this excuse cannot extend to others, who, having borrowed it of him, have gallopped it most unmercifully and indiscriminately, and ridden over every body and every thing that came in their way. Indeed, this hepatic hobby was such a delightful one, that the public soon got hold of it, and presently became connoisseurs in fecal evacuations: nor was it uncommon to hear females in fashionable life lament their having any liver at all; for such patients as these will always be disposed to believe the greatest incongruities which plausibly account for the disorders that they feel, rather than attribute them to their legitimate source,—the turning of day into night, and night into day. The following case will illustrate these preliminary remarks, and will show what errors may arise from generalizing too much.

Miss F—, on the 1st of last September, applied for a singular affection of her left ankle and foot, which she has had for the last nine months. Her age, judging from her appearance, may be 30. She is of a nervous temperament; her complexion is rather florid; countenance animated, bearing upon the whole the marks of health; and her habits have always been domestic. The following account of her case I learnt from herself. About the latter end of the year 1819, she first began to feel an uneasy sensation in her ankle, but so trifling that she took no account of it, and did not prevent her from using her accustomed exercise. It did not long, however, remain in this quiescent state, but became increased in the evening after her dinner, sometimes accompanied with flushed countenance and palpitation of the heart: in the morning she usually became quite easy, the ankle exhibiting no other appearance of disease than a very trifling swelling. Her bowels were generally regular, and her appetite good. She described this uneasiness as a thrilling or creeping sensation, and sometimes, when it was worse than ordinary, she felt as if a number of needles were pricking her. To her own feelings, likewise, the ankle, during the excitement, was unusually hot, although in reality it was not so; and this morbid or erroneous feeling was increased, if the leg was not placed in the horizontal position. Sometimes her toes, and particularly the great toe, became affected with the same sort of pain. At length the disease became more severe, but still with the same character of commencing in the evening. She was obliged to curtail a portion of her accustomed exercise, and her evenings were spent very uncomfortably in retirement. One circumstance connected with this lady's temperament deserves to be noticed,—which is, that she could never be near the fire for any time without feeling a general uneasiness which she could not describe. Hot and close rooms likewise were always uncomfortable to her. This I conceive to be a mark of delicacy, particularly of the nervous system, having remarked similar peculiarities in delicate females who were highly susceptible to nervous impressions.

Her foot and ankle still becoming worse, she was induced to apply to a surgeon of the first eminence in London. He told her that her disease was constitutional, arising from a disordered state of the digestive organs, particularly of the liver; and he desired her to take three grains of mercury with chalk, every second night. This prescription she regularly followed for three weeks, without the slightest benefit: on the contrary, her bowels having been unusually confined during this time, she became more than ordinarily heated after her dinners, and her ankle and foot more painful and swelled. After having taken twelve of the prescribed powders, she returned to her surgeon again; and, as she was no better, expressed a desire that he should examine her leg. This he declined, being convinced that the affection there was merely symptomatic; and desired her to continue her powders as before. She took six more of them; when, not finding any amendment, she applied to another surgeon, who coincided in the opinion of his predecessor.

A short time after this she went to Bath. The carriage motion occasioned by the journey was torture to her, and increased considerably the heat and thrilling sensations in her ankle and foot. Fomentations of warm water, which she now applied to abate the inflammatory appearance, only served to increase the evil. In Bath, she applied for relief to a surgeon of eminence, who prescribed for her cold lotions and embrocations. Another practitioner of respectability, who was strongly recommended by a female friend, again recurred to the hepatic theory. He told her that all her complaints arose from an obstruction of the liver, which could only be removed by a course of purgatives, rest, and low diet. Although she was not a proselyte to this doctrine, she was anxious to try any means that held out a prospect of relief. She was under the care of this gentleman near two months. His first plan of treatment was active purgatives, abstinence from animal food and wine: but, alas! the obstruction was obstinate; and therefoie, contrary to her better judgment, she was bled twice within the space of a week, and she escaped from a third operation by coming to Clifton.

In July last, she first became a patient of the late Mr. Baynton. At this time her health was suffering from the active treatment she had lately submitted to. She was much weakened, and, consequently, the palpitations and other nervous feelings were much increased. The journey from Bath, and a little subsequent exertion, had dissipated the trifling benefit her ankle and foot had derived from a long course of rest; and her spirits, which had never before forsaken her, were now much depressed. Mr. Baynton's opinion was totally different from his predecessors: he considered that the disease was local, and set about treating it accordingly. He applied plaster-straps to her leg, from the foot to the knee; he enjoined her to perfect rest, and to live as usual with respect to diet, omitting her wine. The straps gave her so much pain, that, after the third day of wearing them, a firm calico bandage was substituted in their stead; and she was desired to rub her ankle with an embrocation composed of one part of Goulard's extract, two parts of soap liniment, and three parts of rose-water. She remained under the care of Mr. Baynton, with the most marked benefit, so far as regards ease, comfort, and restoration of general health, up to the time of his death. The evening recurrence of pain in her ankle rarely troubled her while she wore the bandage with its necessary degree of firmness; but if this were left off for ever so short a time, and the limb suffered to hang down, the usual sensations would return, and not leave her until the bandage was replaced.

The shock occasioned by the suddenness of Mr. Baynton's death, brought back her nervous disorders, and with them the painful sensations of her foot and ankle; her leg also, along the course of the tibia, felt uneasy and sore to pressure. At this time it was that I first saw her. I found her mind extremely agitated by the loss of a person, from whose skill she had confidently expected the restoration of her health. Notwithstanding the long continuance of occasional pain in her ankle and foot, no external marks of disease were perceptible on them, except a very trifling degree of redness and swelling. The veins were not in the least varicose. Her extremities, she said, were generally cold; still fire was always disagreeable to her.

A little reflection on the whole of this case made it obvious to my mind, that either it was a constitutional disease producing local effects, or *vice versa:* consequently, a local disease did exist, to which remedies might be, and had been, applied with the best effect. It appeared to me reasonable that the pain which she felt in the ankle and foot, was occasioned by a morbid action of the cuticular nervous extremities of the part; and to this, I thought, might be attributed all the local affection. Pressure, by compressing the cuticular nerves, and by that means depriving them of a certain portion of their power, had been of considerable service, and might, I conceived, be rendered much more efficacious if the pressure were increased, at the same time that the skin was more firmly supported by a close adherence of the remedial means to its surface. I urged the permanent use of the plaster-strap again; but as, by their irritation, she had formerly experienced much distress, I proposed that they should be made of the soap-cerate, which, as a plaster-bandage, may be applied with an equal degree of firmness as the usual adhesive-strap; whilst it is perfectly free from any irritating quality, and may be removed without injury to the thinnest skin. She was, however, desirous to try the calico bandage only, for a short time longer; and she removed from this place to Cheltenham.

Here she applied to three medical men: the first of whom recommended her to immerse her leg nightly in a saturated solution of salt in hot water;—the second assured her that her disease arose from an affection of the kidneys! and recommended her powders of soda, rhubarb, and columba;—the third was of the same opinion as myself with respect to the plaster-strap; and, as she consented to a trial of the plan, he applied them, according to Mr. Baynton's method, from the toes to the knee. Thus equipped, she returned to Clifton, and experienced, during the journey, the greatest comfort from the additional pressure. A few days' wearing of these straps, however, convinced her that the new irritation which they excited would effectually counteract all the good effects of this plan; and she therefore readily assented to my second suggestion of the soap-cerate. This, indeed, fully answered the most sanguine hopes and expectations of either party. The straps thus prepared were applied firmly, as before, from the toes to the knee, with a calico bandage over the whole; and she was desired to walk as much as she could without producing pain or fatigue.

At the end of a week, when the plasters were removed, she had experienced little or none of her old complaint, although walking to some distance had been daily persevered in, and progressively increased: the straps, likewise, were removed without occasioning any additional irritation. I applied them three times, allowing an interval of a week between each application; when she left this place, with every prospect of a permanent recovery.

I am well aware that, by attributing this affection, and other anomalous diseases of the same kind, to a morbid action of the nervous system, I am adducing an hypothesis, the truth of which may justly be disputed. It is, indeed, of but little moment in practice whether painful local affections, which are neither the consequences of inflammation nor ever produce it, be attributable to morbid nervous action only, or to a languid state of the circulation of the affected part, such as we know is the case in a varicose state of the veins. In both instances firm and regular pressure is the most appropriate remedy, and is as certain of producing effectual relief as the most approved medicine is for any other disease. In the one case, the nervous extremities are blunted as it were by the pressure; in the latter, the weakened veins receive an important support from it, and are thus enabled to carry on the circulation with vigour.

I have at present a patient who long had a painful affection of the knee-joint. This is a stout woman, 45 years of age. The disease commenced without any apparent cause. She complained of acute pain and heat on each side of the patella, extending behind it into the interior of the joint. External pressure likewise was painful to her; while rotating the leg, and pressing the articulatory surfaces together, gave her no uneasiness. All that could be seen externally were a few tortuous cutaneous veins, a little enlarged, on the inside of the knee,—such as are generally seen as the commencement of white swelling. The pain was always worse when she was at rest; and was sometimes so considerable at night, when in bed, as to induce her to get up and walk about. Leeches, blistering, cold lotions, and fomentations, gave her but temporary relief: the pain and uneasiness returned again, as soon as the influence of these remedies was gone off. A large varicose vein which now for the first time made its appearance on the leg, determined me to try the effect of pressure. A series of plaster-straps, made with the soap-cerate, was applied, firmly commencing from the lower end of the varicose vein, and extending upwards so as to include the whole of the knee-joint; over this a calico bandage was likewise as firmly applied as possible. The relief which she received was as great as the most sanguine expectation could anticipate. So long as by these means the pressure is kept up, she has no pain in the knee or leg, and she is able to perform the laborious occupations of cook in a large family, with ease and comfort. Whether or not the cure will be permanent, I am not at present able to determine, as she has not ventured to leave off the pressure; but appearances decidedly indicate that it will be so. It is but right to say that the bandage alone, which was fairly tried before the straps were had recourse to, did not produce any alleviation of the disease.

The following cases, showing the good effect of firm pressure in herpes phlyctænodes affecting the leg, being intimately connected with the foregoing subject, may with propriety be inserted here.

Mrs. T—, by falling from a chair on which she was standing, wrenched her knee, which subsequently occasioned a good deal of pain, swelling, and inflammation. The usual remedies were applied, and lastly blisters. Two days after the removal of the last blister, (namely, Sept. 1, 1819,) the vesicated surface became greatly inflamed, hot, and very painful. Poultices, fomentations, rest, and purgatives, were prescribed. On the third, phlyctænæ in patches make their appearance, surrounded by a deep erysipelatous areola. The skin was thickened, unyielding, and glossy. In spite of saturnine and mercurial lotions, ointments of various kinds, and mild mercurial purgatives, the disease spread rapidly around the knee and down the leg, producing, by a constant succession of eruptions, numerous deep, cellular, or honey-comb, ulcers, several of which were large enough to receive the top of the finger, accompanied with a discharge of thin ichrous matter. Beside the increase of heat, and the intolerable itching of the part, which usually came on worse at night, she felt a constant dull pain in the tibia, which gave her the sensation of something gnawing it. On the 27th of September, I determined on trying the effect of pressure. A series of plaster-straps, beginning from the middle of the leg, was applied upwards to the knee, and drawn as tight as possible; over these a calico bandage was also applied, from the foot upwards; the ulcers having been previously washed with a zinc lotion. The pain consequent on this treatment was fully compensated by the comparative ease which she felt after it was accomplished. She was desired to walk about now and then; a luxury she had not dared to indulge in for the last four weeks. This plan of treatment was daily renewed, until the 14th of October, when the ulcers were entirely healed, and have ever since continued so.

About twelve months since I met with another case of herpes, affecting also the upper part of the leg. This was in a woman, aged 43, of an unhealthy bilious habit. She had been suffering for some days before I saw her, and had applied constantly a Goulard lotion to the part, without any benefit. The disease was precisely the same as the one just narrated, and gave way, without any difficulty, to the same plan of treatment.

*Clifton; April* 8, 1821.

